# The impact of adjuvant ultrasound-guided periulcer foam sclerotherapy on venous leg ulcer recurrence: Long-term outcomes

**DOI:** 10.1016/j.jvsv.2026.102537

**Published:** 2026-05-26

**Authors:** Rashad A. Bishara, Ahmed Gaweesh, Ihab Nabil Hanna, Ahmed K. Allam, Mohamed R. Moabed, Sherif Essam, Wassila Taha, Alun H. Davies, Joseph Shalhoub

**Affiliations:** a Organization for Teaching Hospitals & Institutes of Egypt, Cairo, Egypt; b Vein Clinics Egypt, Cairo, Egypt; c Vascular Surgery Department, Alexandria University, Alexandria, Egypt; d iVein Clinic, Alexandria, Egypt; e National Institute for Diabetes and Endocrinology, Cairo, Egypt; f Department of General Surgery, Benah University Hospitals, Benha, Egypt; g Department of Vascular Surgery, Ain Shams University, Cairo, Egypt; h Section of Vascular Surgery, Department of Surgery & Cancer, Imperial College London, London, UK; i Imperial Vascular Unit, Imperial College Healthcare NHS Trust, London, UK

**Keywords:** Venous leg ulcer, Foam sclerotherapy, Distal reflux, Periulcer foam sclerotherapy, PUFS

## Abstract

**Objective:**

The addition of ultrasound-guided periulcer foam sclerotherapy (PUFS) for the incompetent network of veins in the vicinity of a venous leg ulcer (VLU) has been shown to decrease the time to ulcer healing. Long-term follow-up was undertaken to test the hypothesis that PUFS decreases VLU recurrence.

**Methods:**

All patients included in the previously published randomized controlled trial were followed for a minimum period of 12 months after VLU healing to assess VLU recurrence, quality of life (Revised Venous Clinical Severity Score, Short Form-12), and duplex ultrasound findings.

**Results:**

Six of 65 patients were lost to follow-up (1 death). The remaining 58 patients were followed for a minimum of 12 months (mean, 2.2 years). Ulcer recurrence was significantly lower in group A (3/25 [12%]), who received PUFS, compared with group B (15/33 [45%]; *P* = .006). Multivariable Cox proportional hazards regression model showed that group A had a significantly lower hazard of recurrence compared with group B (adjusted hazard ratio, 0.20; 95% confidence interval [CI], 0.06-0.75; *P* = .016). The mean ulcer-free time for group A (1059 days; 95% CI, 960-1158 days) was significantly greater than group B (736 days; 95% CI, 587-885 days; *P* = .002). The Revised Venous Clinical Severity Score was significantly lower in group A (4.45) than in group B (7.94; *P* = .018) at the final follow-up. Patients who had duplex ultrasound examination-confirmed subulcer vein obliteration at follow-up had a significantly improved ulcer-free time (log-rank test, χ^2^ = 9.045; *P* = .003).

**Conclusions:**

The use of adjuvant PUFS decreased the recurrence rates of VLU during a mean follow-up of 2.2 years, increased ulcer-free time, and improved the quality of life. Duplex ultrasound examination-confirmed obliteration of the refluxing subulcer network of veins on follow-up was associated with a lower VLU recurrence.

**Article Highlights**

• **Type of Research:** Prospective randomized controlled trial
• **Key Findings:** Fifty-eight patients were followed for a minimum of 12 months. Ulcer recurrence was significantly reduced in group A (12%), who received periulcer foam sclerotherapy, compared with group B (45%). The mean ulcer-free time for group A (1059 days) was significantly greater than for group B (736 days).
• **Take Home Message:** The use of adjuvant periulcer foam sclerotherapy decreased the recurrence rates and increased ulcer-free time of venous leg ulcers.

Venous leg ulcers (VLUs) are a widespread clinical issue and remain a significant public health concern. As the most advanced stage of chronic venous disease, VLUs are estimated to affect approximately 1% of adults in Western populations, with the prevalence increasing to ≤4% among individuals aged >65 years.^1^ The economic impact is considerable: VLUs consume an estimated 1% to 2% of health care budgets, largely due to the intensive nursing care required. In the UK, for example, treating a patient with a VLU may cost >$10,032 each year, and cases where ulcers do not heal result in costs that are more than four times higher than those for healed ulcers.^2^

In addition to financial strain, VLUs can severely diminish patients' quality of life (QoL). Individuals may experience pain, reduced mobility, sleep disruption, social withdrawal, and mental health challenges such as anxiety and depression. One of the most difficult aspects for patients is the high likelihood of recurrence: studies report that 50% to 70% of VLUs return within 1 year of healing.^3^ This chronic cycle of healing and relapse often means patients contend with the condition for much of their lives.

To address this persistent problem, the existing literature has identified several key factors related to both ulcer management and recurrence prevention.

• Compression therapy remains the primary approach for both treating VLUs and preventing recurrence, because it helps to decrease venous hypertension. However, adherence to compression therapy is frequently suboptimal.^4^
• Surgical options, including procedures to correct superficial venous reflux, have demonstrated reduced recurrence rates when combined with compression, as evidenced by the ESCHAR trial (Effect of Surgery and Compression on Healing And Recurrence).^5^
• Patient self-care strategies—such as elevating the legs, engaging in regular movement and ankle exercises, maintaining a healthy weight, and practicing good skin hygiene—can help to lower the risk of ulcer recurrence.^6^
• Risk factors for VLU recurrence include previous deep vein thrombosis, a history of multiple ulcers, prolonged healing periods for earlier ulcers, and residual superficial venous reflux that remains untreated.^7^

Despite the established efficacy of compression therapy and surgery, high recurrence rates highlight the need for further exploration of adjunctive therapies and better patient adherence strategies.^7^ This context provided the basis for investigating additional methods, such as periulcer foam sclerotherapy (PUFS). We have previously demonstrated the efficacy of PUFS in promoting VLU healing^8^; however, its long-term effect on VLU recurrence has yet to be reported.

## Objectives

This study was conducted to test the hypothesis that the addition of PUFS to standard therapy decreases VLU recurrence. The primary objective was to report the effect of the addition of PUFS to standard therapy on the recurrence of VLU over a minimum follow-up period of 12 months. Secondary objectives are to report—over a minimum follow-up period of 12 months—the effect of PUFS on ulcer-free time, QoL, and duplex ultrasound examination of periulcer veins.

## Methods

The trial was conducted following the principles of the Declaration of Helsinki and was approved by the ethical committee of the General Organization of Teaching Hospitals and Institutes of Egypt. Patient's consent was obtained.

The patients initially included in the previously reported randomized controlled trial (RCT)^8^ were recalled for a follow-up visit. Therefore, patients included in this study are the same patients reported in the previously published RCT.^8^ The follow-up visit was scheduled ≥12 months after complete leg ulcer healing.

Briefly, the multicenter RCT randomized 65 patients to two groups: group A (29 patients) received ultrasound-guided foam sclerotherapy of the refluxing venous plexus underneath and in the vicinity of the ulcer plus standard care, and group B (36 patients) received standard care alone. All patients in group A were treated with one session of ultrasound-guided foam sclerotherapy, except one, who had a second session. The Tessari method was used to make foam using either air (12 VLUs) or carbon dioxide (17 VLUs) with a ratio of liquid to gas of 1:4. Incompetent perforators were not targeted; however, treatment with PUFS occluded incompetent perforators in some cases. After foam sclerotherapy, compression bandages were applied, with ongoing compression as a part of the standard therapy. The management of axial reflux was the same in both groups, with thermal ablation being performed for above-knee great saphenous vein reflux.

The follow-up visits encompassed the following.

• Clinical examination: Physical examination of the VLU site, including photographic documentation.
• Data collection: Recording the date of ulcer recurrence (if applicable) and documenting any use of venotonic drugs or statins.
• Outcome scoring: Administration of the Revised Venous Clinical Severity Score (rVCSS)^9^ and the Short Form 2 (SF-12) QoL questionnaire.^10^ Patients who could not attend the follow-up visit in person underwent a telephone interview, allowing them to be included in the rVCSS and the QoL SF-12.^11^
• Duplex imaging: A duplex ultrasound scan of the ulcer area was performed to determine the status of the refluxing network of veins and any incompetent perforators in the vicinity of or underlying the ulcer. One of the co-authors (W.T.) is an angiologist and an expert in vascular ultrasound examinations who performed most of the duplex ultrasound scans. A fixed-protocol duplex ultrasound scan was used in accordance with standard guidelines.^12^
• Duplex criteria.

Pathological reflux was defined using specific criteria.

• Ulcer vein network: Reflux in the subulcer vein network was considered pathological if it showed bidirectional flow
• Incompetent perforators: Reflux was considered pathological if the outward flow time was >0.5 seconds with a diameter of >3.5 mm.^13^

For patients who were unable to attend the physical visit and unable to complete a duplex ultrasound scan due to long distances, a telephone interview was conducted. These patients were asked to provide a photo of the healed ulcer area or, if present, a photo of the recurrent ulcer.

### Statistical analyses

All analyses were conducted using SPSS version 28 (IBM), Figures were generated using R software (The R Foundation for Statistical Computing). Continuous variables were assessed for normality using visual assessment of histograms and the Shapiro-Wilk test. Normally distributed variables were summarized as mean ± standard deviation and compared using the independent-samples *t*-test. Non-normally distributed variables were expressed as median and interquartile range and compared using the Mann-Whitney *U* test. Categorical variables were presented as frequencies and percentages and compared using the χ^2^ test.

The primary outcome was ulcer recurrence. Recurrence-free survival (ulcer-free time) was analyzed using the Kaplan-Meier method, with comparisons between treatment groups performed using both the log-rank (Mantel-Cox) test and the Breslow (generalized Wilcoxon) test to account for potential early differences in recurrence timing. The mean ulcer-free time for each group was estimated from the Kaplan-Meier survival function.

To identify predictors of recurrence, a multivariable Cox proportional hazards regression model was constructed. Hazard ratios (HRs) and 95% confidence intervals (CIs) were reported. The proportional hazards assumption was checked using log-minus-log survival plots.

For QoL outcomes, within-group changes in the SF-12 Physical Component Summary (PCS) and Mental Component Summary (MCS) scores across the three timepoints (before treatment, after healing, and at the final follow-up) were assessed using the Friedman test. Between-group comparisons at each timepoint were conducted with the Mann-Whitney *U* test. Differences in the rVCSS between groups at the final follow-up visit were compared using the Mann-Whitney *U* test. Among the subset of patients who underwent duplex ultrasound imaging at follow-up, the association between duplex ultrasound examination-confirmed obliteration of the refluxing subulcer vein network or incompetent perforators and ulcer-free time was assessed using Kaplan-Meier curves and the log-rank test. Additionally, Cox regression models were used to estimate unadjusted HRs for recurrence according to duplex findings. A two-sided *P* value of <.05 was considered statistically significant.

## Results

Sixty-five patients were previously randomized into two groups: group A (29 patients) received PUFS plus standard therapy, and group B (36 patients) received standard therapy alone.^8^ No significant differences were observed between the groups in terms of demographic factors, risk factors, history of deep vein thrombosis, previous venous interventions, and ulcer size. There was no significant difference between the two groups in terms of prerandomization duplex ultrasound findings (Table I).^8^

**Table:** Comparison of demographic factors, ulcer characteristics, history of deep vein thrombosis, history of venous interventions, risk factors, and prerandomization duplex ultrasound findings between groups A and B

	Group A, foam sclerotherapy (n = 29)	Group B, no foam sclerotherapy (n = 36)	*P* value
Male sex	20 (69%)	24 (67%)	.84
Mean age, years	46.97 ± 14.43	48.22 ± 14.90	.73
No. of ulcers	Mean, 1.2 (83% had 1 ulcer)	Mean, 1.39 (72% had 1 ulcer)	.60
Ulcer size cm^2^	Median, 3.15 (IQR, 3.75)	Median, 6 (IQR, 9.66)	.10
Ulcer duration, months	Median, 4.0 (IQR, 4.0)	Median, 6 (IQR, 4.5)	**.02**
History of deep vein thrombosis	13 (45%)	12 (33%)	.34
Previous venous intervention	13 (45%)	12 (33%)	.89
Diabetes mellitus	3 (10%)	3 (8%)	.78
Body mass index, kg/m^2^	Median, 29.1 (IQR, 10.5)	Median, 34.7 (IQR, 9.8)	.60
Duplex superficial GSV/SASV/AASV reflux	11 (38%)	18 (50%)	.23
Duplex superficial SSV reflux	10 (34%)	12 (35%)	.92
Duplex extra-axial varicose veins	11 (38%)	15 (42%)	.75
Duplex iliac vein obstruction	3 (10%)	1 (3%)	.20
Duplex infrainguinal PTS	14 (48%)	12 (33%)	.22
Duplex incompetent ulcer veins or incompetent pathological perforator	29 (100%)	36 (100%)	-

*AASV*, Anterior accessory saphenous vein; *GSV*, great saphenous vein; *IQR*, interquartile range; *PTS*, post-thrombotic syndrome; *SASV*, superficial accessory saphenous vein; *SSV*, small saphenous vein.

Number of ulcers: group A 83% had one ulcer; group B 72% had one ulcer (*P* = .6).

Six patients were lost to follow-up, and one patient from group A died of an unrelated cause 4 months after PUFS. The remaining 58 patients, 25 from group A and 33 from group B, were followed for a minimum of 12 months each. The mean follow-up period, calculated from complete ulcer healing until recurrence or the final visit, was 803 days (2.2 years), with a minimum follow-up of 382 days.

### Ulcer recurrence

**Fig 1:**
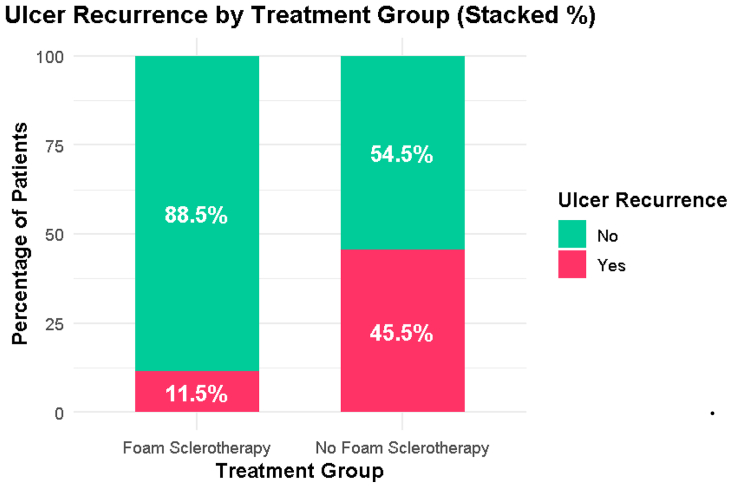
The ulcer recurrence rate was significantly greater in group B compared with group A (Fisher’s exact test *P* = .006).

The ulcer recurrence rate was significantly reduced in group A (3/25 [12%]), which received PUFS, compared with group B (15/33 [45%]; *P* = .006) (Fig 1). Multivariate Cox regression analysis was used to show predictors of ulcer recurrence. Group A had a significantly lower hazard of recurrence compared with group B (adjusted HR, 0.20; 95% CI, 0.06-0.75; *P* = .016), indicating an approximately 80% decrease in hazard. Other variables, including sex, age, body mass index, and ulcer area, were not significantly associated with ulcer recurrence (Supplementary Table I, online only). The mean time to ulcer recurrence was longer in group A (638.8 days) compared with group B (357 days). Kaplan-Meier curves for time to recurrence showed a statistically significant difference between group A and group B (log-rank test *P* = .0039) (Supplementary Fig 1, online only).

### Ulcer-free time

**Fig 2:**
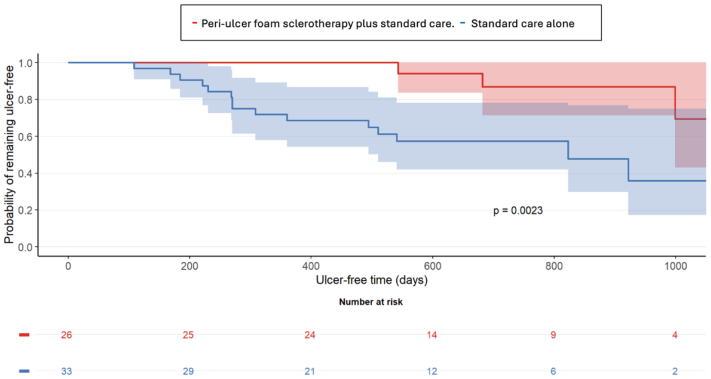
Kaplan-Meier curves indicate a statistically significant difference in ulcer-free time between the two treatment groups—group A, which was treated with periulcer foam sclerotherapy (PUFS) plus standard care, and group B, which was treated with standard care alone. Log-Rank test *P* = .002.

The ulcer-free time was calculated using the Kaplan-Meier curve, and the difference between the two groups was assessed using the log-rank test and the Breslow test. The mean ulcer-free time for group A (1059.04 days; 95% CI, 960.39-1157.69 days) was significantly greater than group B (735.89 days; 95% CI, 587.21-884.70 days; *P* = .002), suggesting that PUFS plus standard therapy is associated with a longer ulcer-free time compared with standard therapy alone (Fig 2).

### QoL

#### rVCSS

We previously reported that both groups A and B were similar on the rVCSS before treatment and after treatment, and that both groups showed a statistically significant improvement as a result of treatment. Assessment of the rVCSS at the final visit revealed a significant improvement in group A (4.450 ± 4.005), which received PUFS, compared with group B (7.940 ± 6.930; *P* = .018) (Supplementary Fig 2, online only).

#### SF-12 QoL scores

The SF-12, validated in the local language,^10^ was used to assess QoL at three timepoints: before treatment, after treatment at the time of complete ulcer healing, and at the final visit, which occurred at ≥12 months after ulcer healing. Within-group comparisons using the Friedman test showed a statistically significant improvement in the MCS over time in both groups (group A, *P* = .009; group B, *P* = .004). Conversely, the PCS did not show significant differences between groups (*P* > .05). Between-group comparisons using the Mann-Whitney *U* test indicated no statistically significant differences between group A and group B at any timepoint for either PCS or MCS (*P* > .05 for all) (Supplementary Fig 3, online only).

#### Duplex ultrasound findings

**Fig 3:**
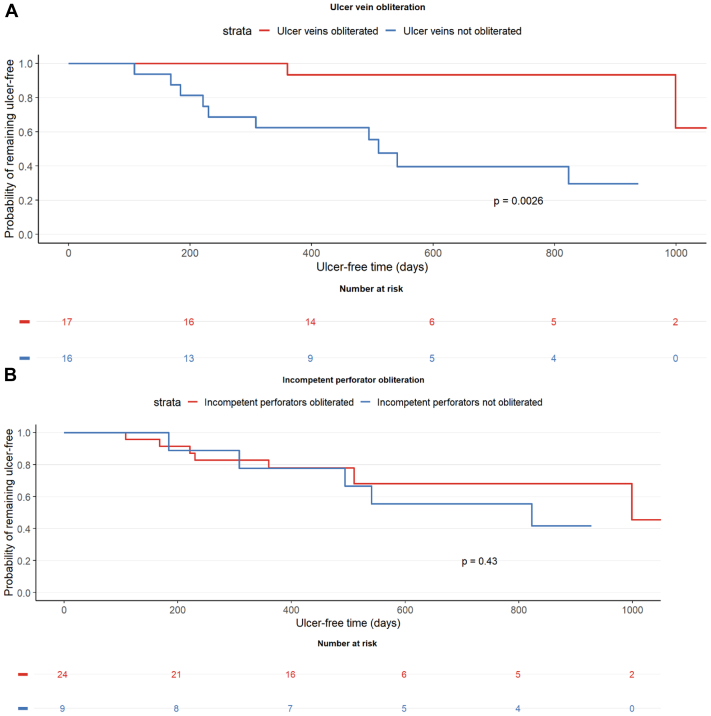
Kaplan-Meier curves showing the association between ulcer vein obliteration and ulcer-free time (*top*) and incompetent perforator obliteration and ulcer-free time (*bottom*). Log-rank *P* = .0026 for ulcer vein obliteration and *P* = .43 for incompetent perforator obliteration.

Only 32 of the 58 patients (55%) underwent a duplex ultrasound examination of the ulcer area. The operator reported the status of the incompetent network of veins in the vicinity of the ulcer and of the incompetent perforators, whether obliterated, partially obliterated, or patent, and, if patent, whether refluxing or not. The log-rank test showed a significant improvement in ulcer-free time between patients with ulcer vein obliteration on follow-up duplex ultrasound examination and those without (χ^2^ = 9.045; *P* = .003). However, the ulcer-free time did not differ significantly between patients with obliterated incompetent perforators and those without in the long-term follow-up (Fig 3 and Supplementary Table II, online only).

#### Compression

The type of compression used was at the discretion of the treating physician. The number of patients treated by compression and the type of compression used are shown in Supplementary Table III (online only). Data regarding adherence to the prescribed compression were collected; however, adherence was not associated with VLU recurrence (Cox regression analysis *P* = .96).

## Discussion

Numerous previous studies have addressed the prevention of venous ulcer recurrence.^14^, ^15^, ^16^, ^17^, ^18^ The ESCHAR trial demonstrated that traditional surgical correction of superficial venous reflux (in addition to compression) significantly decreased ulcer recurrence rates at 4 years. However, it did not demonstrate improved ulcer healing rates.^19^^,^^20^ EVRA (Early Venous Reflux Ablation) showed that early endovenous ablation of superficial venous reflux (in addition to compression) resulted in faster healing of VLUs and more time free from ulcers when compared with deferred ablation.^21^ This trial, however, did not address the issue of ulcer recurrence.

Although the use of foam sclerotherapy to obliterate the periulcer venous plexus was described as early as 2005,^22^^,^^23^ there is ongoing interest in incorporating this technique within therapeutic pathways for individuals with VLUs.^24^

In our RCT, the early results of which have previously been published,^8^ the addition of PUFS to standard care improved treatment outcomes with a significantly shorter time to complete ulcer healing. The long-term results of the previously published RCT, which are reported in this article, highlight PUFS as significantly decreasing the VLU recurrence rate and increasing ulcer-free time. This finding is novel; the effect of PUFS on VLU recurrence in long-term follow-up has not previously been reported. These results, however, are based on a limited sample size and should be interpreted cautiously. PUFS is a simple, inexpensive technique that can be used as an adjunct to the established modalities of care used in VLU management, particularly compression and ablation of truncal reflux, to further decrease VLU recurrence rates.

Unlike previous studies that focused on abolishing truncal reflux or targeting individual perforators,^18^ PUFS aims to obliterate the network of veins in the vicinity of and beneath the ulcer, known as the subulcer venous plexus or ulcer veins.

An important finding of this study is the association between duplex ultrasound examination-confirmed obliteration of the refluxing ulcer veins at follow-up and the lower rate of VLU recurrence. Note, however, that the number of patients who underwent follow-up ultrasound imaging was small (n = 32 of 58). This finding supports the hypothesis that local periulcer pathology is a critical driver of recurrence, beyond the main superficial trunks. In this study, the successful closure of this local venous network, often residual after initial treatment of truncal reflux, is a facilitator of long-term success. In contrast, obliteration of incompetent perforators in the vicinity was not in itself associated with a prolonged ulcer-free time.

The rVCSS,^9^ a comprehensive, disease-specific assessment tool, demonstrated that PUFS provided a sustained and superior clinical outcome beyond initial ulcer healing. However, an assessment of generic QoL showed no significant difference; this was similar to the findings of generic QoL results from EVRA, which used the SF-36 rather than the SF-12.^21^

### Study strengths and limitations

The strength of this study comes from the long-term follow-up of the previously published RCT. The main limitation is patient loss to follow-up (n = 6 of 65), with a final number of 58 patients completing follow-up, of whom only 32 underwent follow-up duplex ultrasound imaging. Other limitations included a lack of standardization for compression and venoactive use and the fact that the rVCSS was not visually evaluated in all patients.

## Conclusions

Adjuvant PUFS can be viewed as a beneficial addition to VLU management, with the findings of this study demonstrating that it may support ulcer healing and help to decrease the risk of VLU recurrence.

## Author Contributions

Conception and design: RB, AD, JS

Analysis and interpretation: RB, MM, AD, JS

Data collection: RB, IH, AA, MM, WT

Writing the article: RB

Critical revision of the article: RB, AG, IH, AA, MM, SE, WT, AD, JS

Final approval of the article: RB, AG, IH, AA, MM, SE, WT, AD, JS

Statistical analysis: Not applicable

Obtained funding: Not applicable

Overall responsibility: Not applicable

## Funding

None.

## Disclosures

None.
