# Supplementary material for: The impact of adjuvant ultrasound-guided periulcer foam sclerotherapy on venous leg ulcer recurrence: Long-term outcomes
Source: J Vasc Surg Venous Lymphat Disord. 2026 May 26;14(5):102537. doi: 10.1016/j.jvsv.2026.102537 (PMC13314928; doi:10.1016/j.jvsv.2026.102537)
Supplement: Supplementary Figures and Tables [file mmc1.docx]

**Supplementary Tables & Figures**

**The impact of adjuvant ultrasound-guided peri-ulcer foam sclerotherapy on venous leg ulcer recurrence. Long-term outcomes.**

| Healing Time in Days | Spearman’s rho | | Kendall's tau_b | |
| --- | --- | --- | --- | --- |
|  | Correlation Coefficient | Sig. (2-tailed) | Correlation Coefficient | Sig. (2-tailed) |
| Age Groups | .005 | .963 | .006 | .96 |
| Gender | -.071 | .496 | -.085 | .50 |
| Number of ulcers | .169 | .096 | .202 | .10 |
| Ulcer area | .241 | **.005** | .349 | **.004** |
| Ulcer duration in months | .139 | .119 | .197 | .11 |
| Weight in Kg | .260 | **.003** | .367 | **.003** |
| Height in meter | .142 | .107 | .196 | .11 |
| BMI | .140 | .105 | .213 | .08 |
| Ulcer depth | .031 | .768 | .03 | .77 |
| Ulcer location | .133 | .193 | .167 | .18 |
| History of DVT | .022 | .834 | .026 | .83 |
| DM | -.111 | .286 | -.133 | .29 |
| Myocardial impairment | -.110 | .287 | -.133 | .29 |

**Supplementary Table 1.** Correlation between healing time and all variables.

| Coefficients ^a^ | | | | | |
| --- | --- | --- | --- | --- | --- |
| Factors | | Unstandardized Coefficients | t | Sig. | Effect on healing time among the groups |
|  |  | B |  |  |  |
| 1 | (Constant) | **1.266** | **10.003** | **.000** | **Significance association** |
|  | study groups | **.235** | **3.031** | **.004** |  |
|  | study groups | .239 | 3.084 | .003 | Non |
|  | Age | -.003 | -1.140 | .259 |  |
| 2 | Study groups | .236 | 3.032 | .004 | Non |
|  | Gender | -.054 | -.652 | .517 |  |
| 3 | study groups | .232 | 2.937 | .005 | Non |
|  | Number of ulcers | .024 | .345 | .731 |  |
| 4 | study groups | .221 | 2.826 | .006 | Non |
|  | Ulcer area | .004 | 1.231 | .223 |  |
| 5 | study groups | .237 | 3.032 | .004 | Non |
|  | Ulcer depth | .076 | .566 | .574 |  |
| 6 | study groups | .229 | 2.924 | .005 | Non |
|  | Ulcer location | .054 | .780 | .439 |  |
| 7 | study groups | .213 | 2.619 | **.011** | **Confounder** |
|  | Ulcer duration in months | .007 | .929 | **.356** |  |
| 8 | study groups | .240 | 3.056 | .003 | Non |
|  | History of DVT | .043 | .537 | .593 |  |
| 9 | study groups | .233 | 2.993 | .004 | Non |
|  | DM | -.110 | -.821 | .415 |  |
| 10 | study groups | .260 | 3.332 | .001 | Non |
|  | Myocardial impairment | -.302 | -1.631 | .108 |  |
| 11 | study groups | .182 | 2.378 | **.021** | **Effect modifier** |
|  | Weight in Kg | .005 | 2.721 | **.008** |  |
| 12 | study groups | .230 | 3.044 | **.003** | **Effect modifier** |
|  | Height in meter | .685 | 2.071 | **.043** |  |
| 13 | study groups | .213 | 2.711 | .009 | Non |
|  | BMI | .007 | 1.395 | .168 |  |
| 14 | study groups | .226 | 2.872 | .006 | Non |
|  | Duplex Superficial GSV/SASV/ AASV Reflux | .062 | .786 | .435 |  |
| 15 | study groups | .236 | 3.035 | .004 | Non |
|  | Duplex Superficial SSV Reflux | .071 | .864 | .391 |  |
| 16 | study groups | .234 | 2.992 | .004 | Non |
|  | Duplex Extra-axial varicose veins | .038 | .484 | .630 |  |
| 17 | study groups | .229 | 2.899 | .005 | Non |
|  | Duplex iliac v. obstruction | -.079 | -.485 | .630 |  |
| 18 | study groups | .235 | 2.968 | .004 | Non |
|  | Duplex infra-inguinal PTS | -.002 | -.028 | .977 |  |
| 19 | study groups | .257 | 3.264 | .002 | Non |
|  | Duplex incompetent ulcer veins | .256 | 1.378 | .173 |  |
| 20 | study groups | .235 | 3.025 | .004 | Non |
|  | Duplex incompetent pathologic perforator | -.066 | -.782 | .437 |  |
| 21 | study groups | .240 | 3.045 | .003 | Non |
|  | Ablation of superficial axial reflux | 8.10 | .62 | .536 |  |

**Supplementary Table 2.** Results of multivariate regression analysis. The dependent variable was healing time.

| **Type of compression** | **Number of patients (percent)** |
| --- | --- |
| Elastic stockings | 28 (50%) |
| Adjustable wraps | 8 (13%) |
| Elastic bandage | 4 (6%) |
| Short-stretch bandages | 2 (3%) |
| No compression | 16 (27%) |

**Supplementary Table 3.** Use of compression within the study.

**Supplementary Figure 1.** Time to complete ulcer healing was significantly shorter in Group A (received peri-ulcer foam sclerotherapy) as compared to Group B (did not receive peri-ulcer foam sclerotherapy): median 35 days, IQR 22 vs median 56 days, IQR 58; p=.008.

**Supplementary Figure 2.** VCSS was assessed initially upon presentation (before TT), and after complete ulcer healing (after TT), and on long-term follow up (FU). There was a significant improvement in Venous Clinical Severity Score (VCSS) for both groups (Group A receiving sclerotherapy and Group B not receiving sclerotherapy) after treatment p<.0001. Group A showed significant improvement on long-term follow up compared to Group B p=0.018. TT = treatment. FU = Follow up.


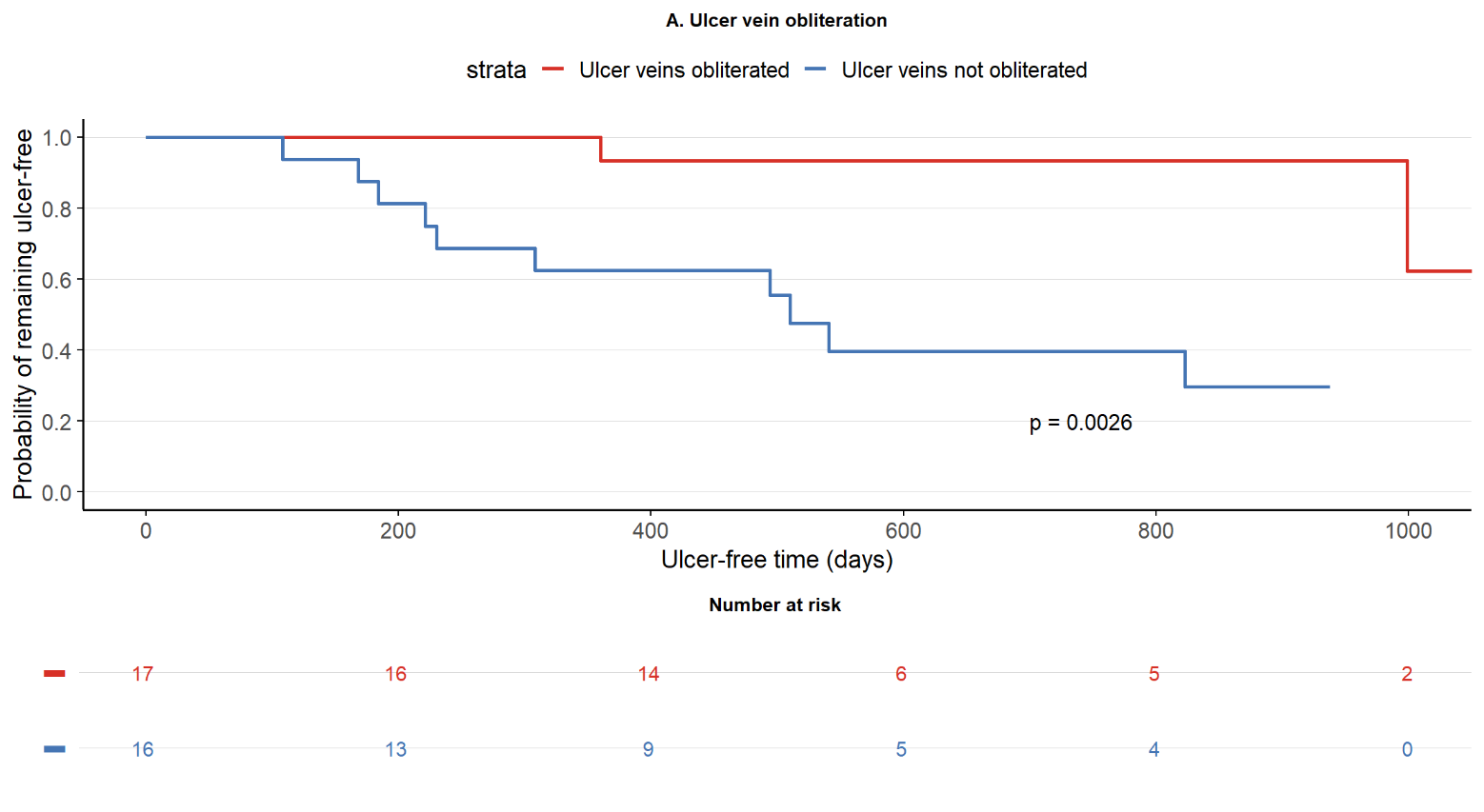


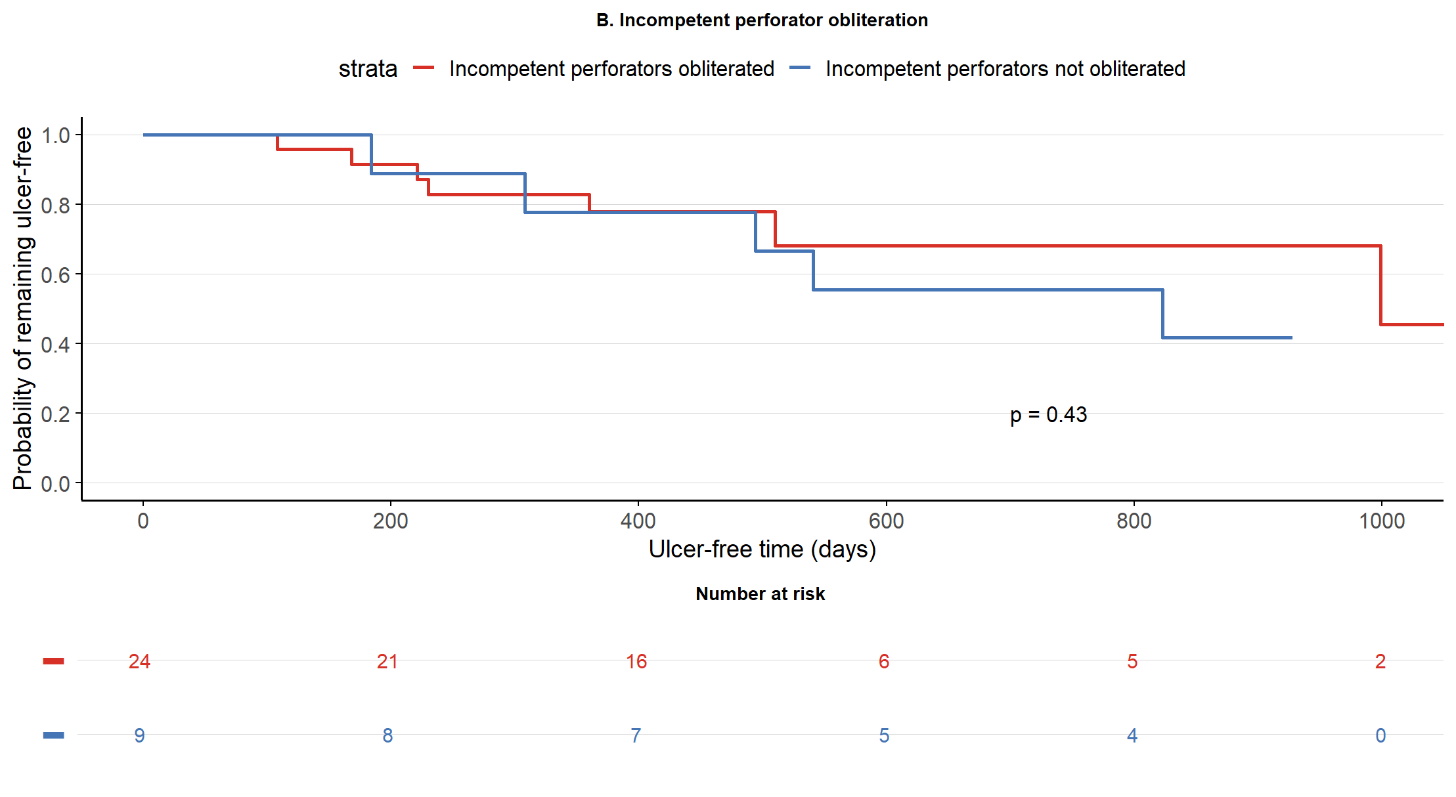


**Supplementary** **Figure 3.** Kaplan–Meier curves showing the association between ulcer vein obliteration and ulcer-free time (top panel) and incompetent perforator obliteration and ulcer-free time (bottom panel). Log-rank p = 0.0026 for ulcer vein obliteration and p = 0.43 for incompetent perforator obliteration.
